# External validation of a prediction model for CPAP failure in COVID-19 patients with severe pneumonitis

**DOI:** 10.1186/s13054-022-04144-4

**Published:** 2022-09-27

**Authors:** Victoria Stokes, Kwee Yen Goh, Graham Whiting, Sebastian Bates, Hannah Greenlee, Anthony Wilson, Alexander J. Parker

**Affiliations:** 1 Adult Critical Care Department, Manchester Royal Infirmary, Manchester University NHS Foundation Trust, Manchester, UK; 2 Research and Innovation, Manchester University NHS Foundation Trust, Manchester, UK

Dear Editor,

Continuous positive airway pressure (CPAP) has been widely used as an intervention to attempt to avoid mechanical ventilation in patients with coronavirus disease 2019 (COVID-19) [1].

Whilst CPAP can be a useful tool, it is recognised that there are a group of patients for whom this treatment is ineffective [2]. Arina et al. demonstrated that critical care admission biomarkers such as CRP and NT-proBNP may identify patients in whom CPAP is likely to fail (resulting in invasive mechanical ventilation (IMV) or death) [3]. We used routinely collected healthcare records of COVID-19 patients treated on the intensive care unit (ICU) at Manchester Royal Infirmary to independently validate their findings. We assessed their model using a sub-cohort treated at the time the model was developed (prior to the introduction of dexamethasone as a routine treatment, 15 June 2020) and also using our entire cohort of patients (1 March 2020 to 31 October 2021) [4]. Supplementary results are available in Additional file 1.

Our entire cohort included 336 patients with confirmed COVID pneumonitis, of whom 215 received at least six hours of CPAP in a 24-h window. 148 (69%) were CPAP failures. For model validation, there were 148 patients with sufficient data of whom 103 (70%) were CPAP failures. In the sub-cohort, there were 32 patients (24 (75%) failures). Additional file 1: Table S1 summarises the patient demographics.

We compared variables measured on ICU admission in CPAP successes and failures and found significant differences in CRP (*p* = 0.001), troponin T (*p* = 0.046), D-Dimer (*p* < 0.001), and age (*p* < 0.001). There was no significant difference in NT-proBNP (*p* = 0.190) or highest respiratory rate in the first 24 h of ICU admission (*p* = 0.417). Figure 1 summarises the difference in biomarkers across the whole cohort, and Additional file 1: Fig. S1 shows the differences in the sub-cohort.

**Fig. 1:**
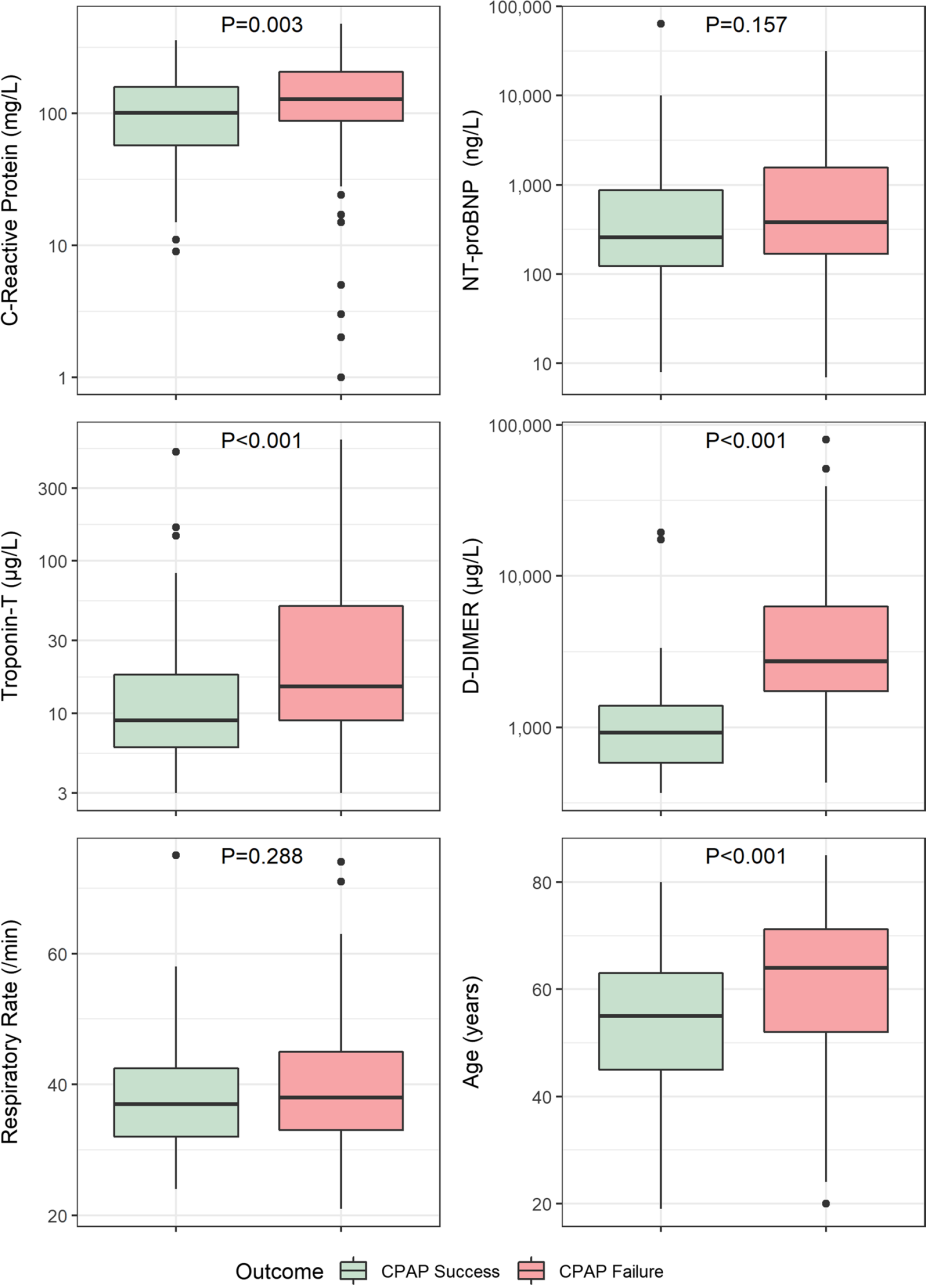
Biomarkers and variables recorded in the COVID population on ICU admission in patients receiving CPAP. *NT-proBNP* N-terminal pro-B-type natriuretic peptide

In our sub-cohort, the model proposed by Arina had an AUC = 0.839. Calibration was acceptable although we observed a slightly higher failure rate than expected (Additional file 1: Fig. S2). The model performed poorly when extended to our entire cohort (AUC = 0.613). Our results suggest that although there is a simple biomarker-based model to predict CPAP failure in ICU patients in the early stages of the COVID-19 pandemic, it is unlikely to be helpful now. Later models have sought to predict CPAP failure by including variables which describe patients’ work of breathing [5].

Since the beginning of the pandemic, a range of factors have modified the course of the disease including vaccination, drug treatments and improved clinical experience. Disease-modifying agents used to treat COVID-19 deserve particular attention and may partly explain why predictive models based on markers of generalised inflammation now perform poorly. Further work is needed to characterise how predictors of CPAP failure in COVID-19 have changed over time. Understanding which patients now have a higher likelihood of CPAP failure may help focus resources and direct preparedness in planning for CPAP failure in this cohort. Further studies to understand the timing and role for mechanical ventilation in this group may assist in further improvements in the management of these challenging patients.

## Supplementary Information

**Additional file 1.** Supplementary figures and tables.

## Data Availability

Not applicable.
