# Supplementary material for: External validation of a prediction model for CPAP failure in COVID-19 patients with severe pneumonitis
Source: Crit Care. 2022 Sep 27;26:293. doi: 10.1186/s13054-022-04144-4 (PMC9513984; doi:10.1186/s13054-022-04144-4)
Supplement: Supplementary file 1 — Additional file 1. Supplementary figures and tables. [file 13054_2022_4144_MOESM1_ESM.docx]

**SUPPLEMENTARY DIGITAL CONTENT**

**EXTERNAL VALIDATION OF A PREDICTION MODEL FOR CPAP FAILURE IN COVID-19 PATIENTS WITH SEVERE PNEUMONITIS**

MRI Critical Care Contributors:

Victoria Stokes^1^, Kwee Yen Goh^1^, Graham Whiting^1^, Hannah Greenlee, ^1^ Andrew Martin^1^, Anthony Wilson^1^, Alexander J Parker^1^, Cathie Forrest^1^, Sebastian Bates^2^

^1^Adult Critical Care Department, Manchester Royal Infirmary, Manchester University NHS Foundation Trust, Manchester, United Kingdom

^2^Research & Innovation, Manchester University NHS Foundation Trust, Manchester, UK.

| Figure 1: | Demographics and Outcomes of Covid Population |
| --- | --- |
| Figure 2: | Biomarker and admission variables for pre-dexamethasone validation cohort |
| Figure 3: | Calibration plot of Arina’s Model |

|  | Full Population | | Pre-Dexamethasone sub-cohort | |
| --- | --- | --- | --- | --- |
|  | CPAP Success N=67 | CPAP Failure N=148 | CPAP Success N=8 | CPAP Failure N=24 |
| Age (years) | 55.0 [45.0-63.0] | 64.0 [52.0-71.2] | 50.0 [44.5-57.0] | 62.5 [51.2-71.0] |
| Male | 39 (58.2%) | 97 (65.5%) | 8 (100%) | 16 (66.7%) |
| Body Mass Index | 31.6 [27.4-38.9] | 28.9 [26.1-35.6] | 27.9 [26.8-28.4] | 28.2 [25.9-34.0] |
| Time on ICU (days) | 6 [5-9] | 14 [6-25] | 5.0 [3-8] | 11 [5-22] |
| CPAP duration (hours) | 34 [17-67] | 44 [20-83] | 32 [25-49] | 34 [15-81] |
| Intubated | 0 | 102 (68.9%) | 0 | 15 (62.5%) |
| Died | 0 | 109 (73.6%) | 0 | 17 (70.8%) |

**Supplementary Table 1** Demographics and outcomes. Data shown as n (%) or median [IQR]. Pre-dexamethasone sub-cohort n=44, 12 patients excluded due to incomplete data.


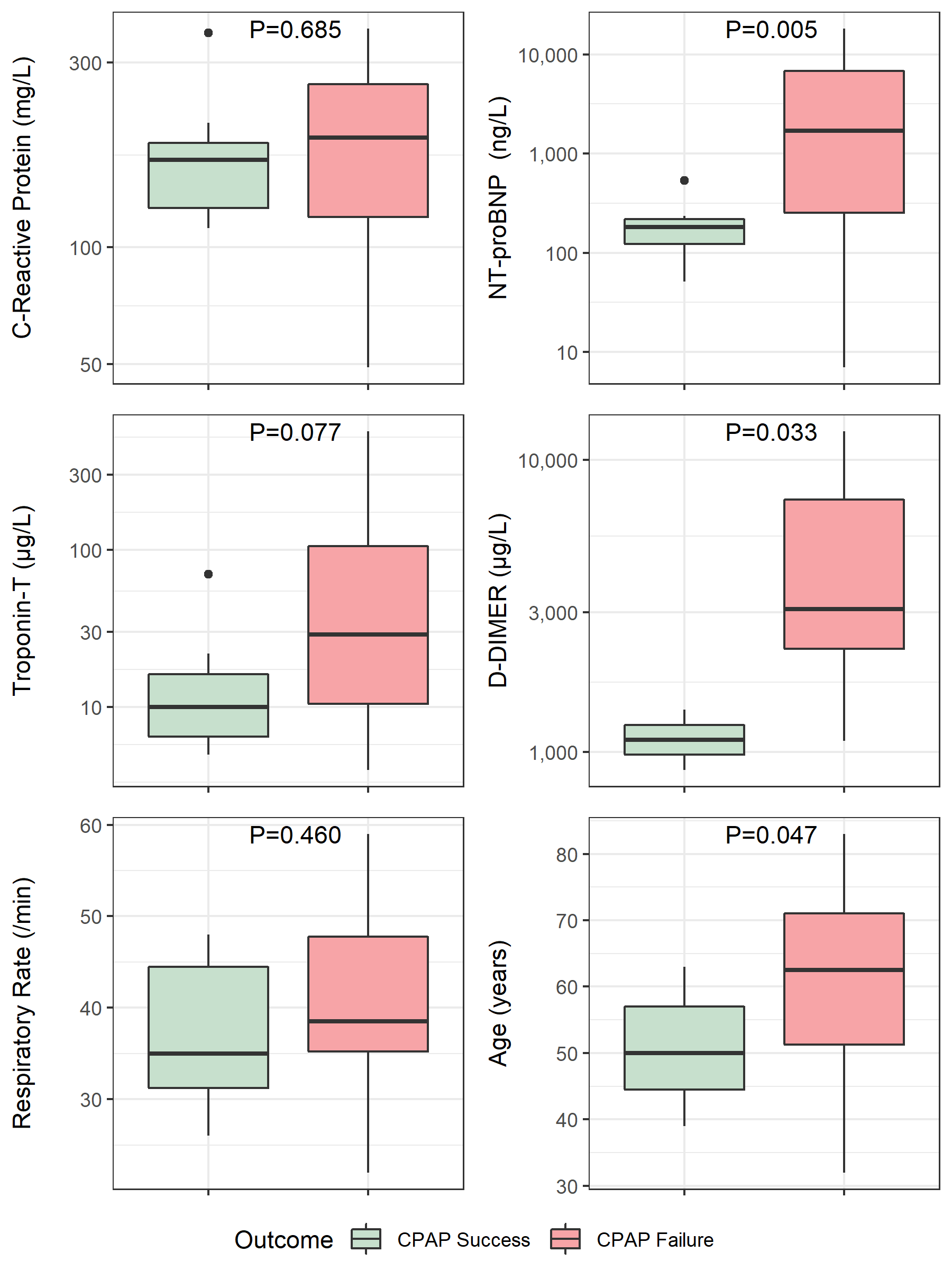


**Supplemental Fig. 1** Biomarkers and variables recorded on ICU admission in patients receiving CPAP in the pre-Dexamethasone validation cohort. *NT-proBNP* N terminal-pro B-type natriuretic peptide


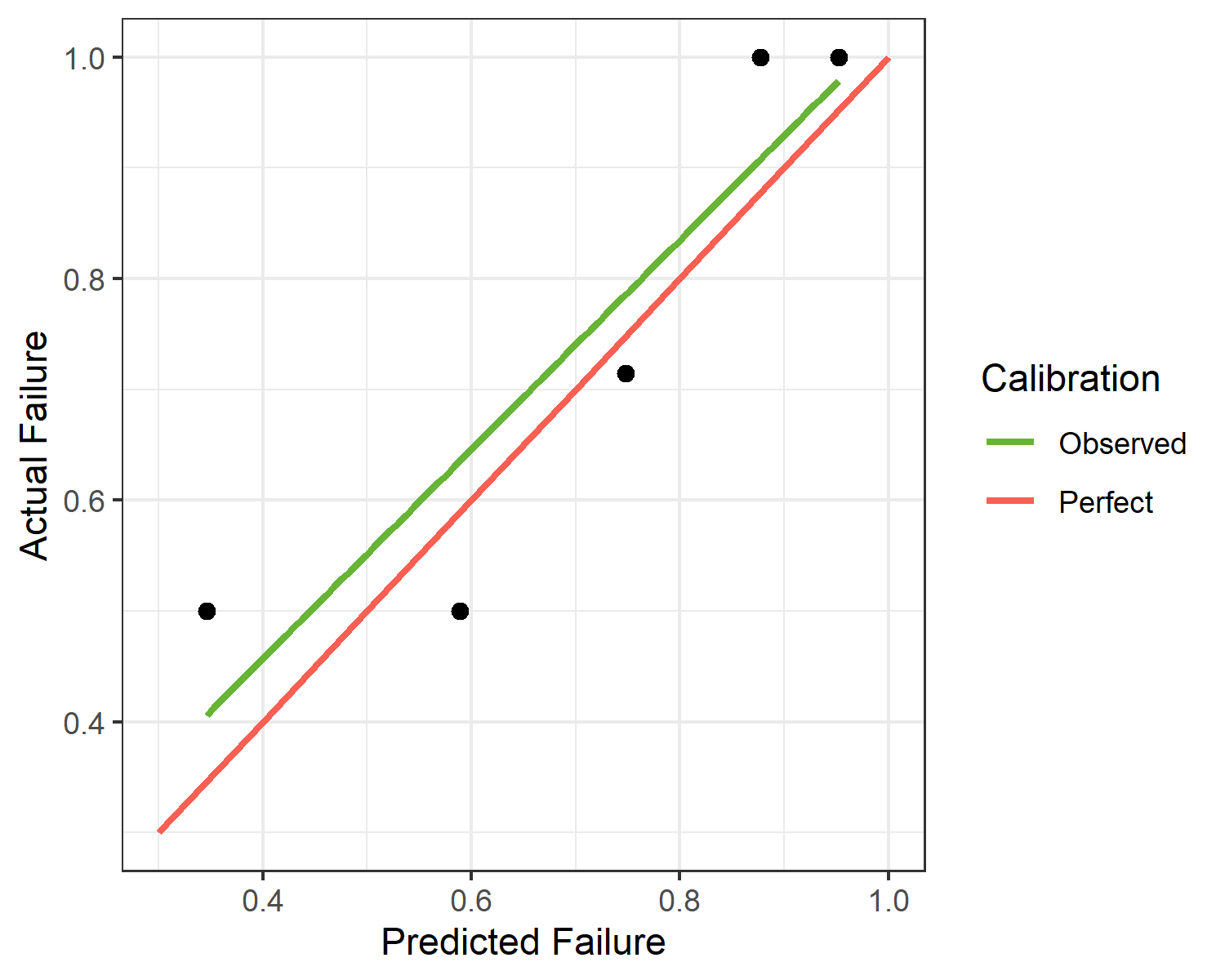


**Supplemental Fig. 2** Calibration plot of Arina’s model. Risks are groups in quintiles
